# HOG-SVM Impurity Detection Method for Chinese Liquor (Baijiu) Based on Adaptive GMM Fusion Frame Difference

**DOI:** 10.3390/foods11101444

**Published:** 2022-05-17

**Authors:** Xiaoshi Shi, Zuoliang Tang, Yihan Wang, Hong Xie, Lijia Xu

**Affiliations:** 1 College of Mechanical and Electrical Engineering, Sichuan Agricultural University, Ya’an 625014, China; shixiaoshi@stu.sicau.edu.cn (X.S.); zuoliang_tang@stu.sicau.edu.cn (Z.T.); 2020217014@stu.sicau.edu.cn (Y.W.); xiehong@stu.sicau.edu.cn (H.X.); 2 College of Resources, Sichuan Agricultural University, Chengdu 611130, China

**Keywords:** bottled Baijiu, histogram projection, Gaussian mixture model, fame difference method, HOG features, SVM

## Abstract

Chinese liquor (Baijiu) is one of the four major distilled spirits in the world. At present, liquor products containing impurities still exist on the market, which not only damage corporate image but also endanger consumer health. Due to the production process and packaging technologies, impurities usually appear in products of Baijiu before entering the market, such as glass debris, mosquitoes, aluminium scraps, hair, and fibres. In this paper, a novel method for detecting impurities in bottled Baijiu is proposed. Firstly, the region of interest (ROI) is cropped by analysing the histogram projection of the original image to eliminate redundant information. Secondly, to adjust the number of distributions in the Gaussian mixture model (GMM) dynamically, multiple unmatched distributions are removed and distributions with similar means are merged in the process of modelling the GMM background. Then, to adaptively change the learning rates of the front and background pixels, the learning rate of the pixel model is created by combining the frame difference results of the sequence images. Finally, a histogram of oriented gradient (HOG) features of the moving targets is extracted, and the Support Vector Machine (SVM) model is chosen to exclude bubble interference. The experimental results show that this impurity detection method for bottled Baijiu controls the missed rate by within 1% and the false detection rate by around 3% of impurities. Its speed is five times faster than manual inspection and its repeatability index is good, indicating that the overall performance of the proposed method is better than manual inspection with a lamp. This method is not only efficient and fast, but also provides practical, theoretical, and technical support for impurity detection of bottled Baijiu that has broad application prospects.

## 1. Introduction

Baijiu is one of the four main distilled spirits in the world [1,2]. As of December 2020, the Baijiu production has reached 54.74 million kilolitres [3] with a year-on-year increase of 9.06% [4]. Although the Baijiu industry is thriving, there are numerous problems with the quality in the process when filling bottled Baijiu [5]. There still exist bottled Baijiu products on the market which contain impurities, and they damage the corporate image and endanger consumer health, which is a serious food safety issue. The State Food and Drug Administration of China sampled 3000 batches of bottled Baijiu, and the filling failure rate was up to 9.26% with 278 collections of filling substandard products. Impurities usually appear in bottled Baijiu due to the production process and encapsulation techniques, such as glass debris, mosquitoes, aluminium scraps, hair and fibres. So, it is essential to implement strict impurity detection for bottled Baijiu products [6]. At present, it mainly relies on manual lamp inspection to check the filling quality of the bottled Baijiu productions in China. According to statistics, it takes 5–10 s to manually inspect a bottle of Baijiu, with an average error rate of about 5% and an average missing rate of about 2%. In the face of rising sales of Baijiu, there is a very urgent need for Baijiu companies to introduce a fast, accurate, and low-cost impurity detection method to improve efficiency and to realise the autonomous and intelligent inspection process of bottled Baijiu.

In recent years, Seidenada (Germany), Brevetti (Italy), and Eisai (Japan) have developed some types of equipment for impurity detection. The equipment developed by Seidenada uses a tracking vision inspection solution. The main principle of it is to rotate the product to be inspected at a high speed and then stop sharply, during which the light path is adjusted according to the rotation control of the reflector, and the fixed camera acquires a sequence of images. According to those sequences of images, it can be discerned whether there are moving impurities in the product or not. The equipment developed by Brevetti uses an intermittent vision inspection solution. The main principle of it is to put the products that are to be inspected through a high-speed rotating station and pause for *n* milliseconds when arriving at the photo point so that the camera can take a sequence of images. Only if there is a need for the industrial camera to take pictures, the rotating station will stop, so the images acquired by this solution are very stable and clear. Its mechanical structure is relatively simpler than the tracking type aforementioned, but its moving speed is slower. As the inertia of the inspection target is usually large, the vibration of the target in this way will be strong when stopped sharply, so it is not suitable for the application to large-capacity bottled products. The equipment developed by Eisai uses a photosensitive sensor to detect moving foreign objects with the light beam shining directly through the liquid onto an array of photodiodes on the other side of the photosensitive head. If any particles are moving in the liquid, the light beam will change and form a changing shadow resulting in a corresponding change in the light on the photosensitive sensor. The sensor can recognise the shadow and convert it into electrical signals, and it can be determined whether there are foreign bodies in the container by judging the electrical information. The pieces of the equipment described above are mainly used in foreign object inspection for bottled pharmaceutical reagents and fizzy drinks, and very rarely used for bottled Baijiu. However, the experience from the above research of impurity detection in other industries instead of Baijiu is still relevant.

In 1991, Huang [7] proposed a method to detect impurities in liquids. In this method, a bottle filled with liquid is rotated at a high speed and then suddenly stopped to facilitate the capture of images of the impurities. This method has been widely used since. In 2002–2004, Kato [8] and Shimizu [9] studied the space and time properties of impurity, and they proposed a method that could identify the size and shape of tiny particles in bottled mineral water to achieve impurity detection. In 2014, Huang [10] designed a detection system that used least-squares filtering for image noise suppression and treated impurity as closed domains of a specific size in images. In 2019, Diaz [11] proposed a detection method by using high-frequency measurement sensors. In this method, the transmitting and receiving signals of those high-frequency sensors are used to measure changes in the frequency of the liquid level, and the results will be used to identify the filling quality of bottled products. Li [12] proposed a real-time detection system for tiny impurities in transparent bottled liquid based on machine vision, which combined roundness calculation, longitudinal frame difference, orthogonal axis detection and KNN (K-Nearest Neighbor) algorithm. Zhou [13] investigated the combination of two-difference and energy accumulation methods for the detection of impurities in liquid and proposed an adaptive filtering algorithm for avoiding noise interference to better inspect small moving targets. Cano [14] researched the detection technology of impurities in olive oil and extracted the features of impurities in the CIELaB and HSV (Hue–Saturation–Value) spaces through the histogram of the colour image. In this method, the SVM and the ANN are used to detect the impurities.

In the process of detecting impurities in bottled Baijiu, there is often interference from surface stains on the bottle. So, the extraction of impurities of bottled Baijiu is usually transformed into a motion target detection problem for a better result. The commonly used methods of motion target detection are the frame difference method [15,16,17], the optical flow method [18,19], and the background subtraction method [20,21,22]. According to previous research results, a single traditional algorithm does not achieve good detection results. Kanagamalliga [23] calculated the field of the optical flow method to carry out the moving target detection according to the optical flow distribution characteristics. But this method is computationally intensive resulting in slow detection. Shu [24] proposed an application of the traditional frame difference method to track a moving target, which was fast but the detection results obtained had a Hole phenomenon. Tang [25] treated flames as moving targets and extracted the foreground of images using the Gaussian mixed model (GMM). GMM has a high recognition rate for moving targets, but it uses a fixed learning rate and a fixed number of Gaussian distributions which leads to the existence of afterimages directly.

Considering the analysis mentioned above, this paper proposes a flip mechanism which can promote the movement of impurities and a novel detection method for moving impurities in bottled Baijiu to overcome the problem caused by the substantial diversity of impurities in bottled Baijiu and the different moving speeds of impurities. The adaptive GMM fusion frame difference with the adjustable number of Gaussian distributions and the learning rate is firstly proposed to segment moving targets in this paper. In our method, the support vector machines (SVM) are used to detect impurities in bottled Baijiu according to those features after extracting the histogram of oriented gradient (HOG) features of the moving target. Our novel method has four main steps. Firstly, an image acquisition system is built to obtain high-quality images of bottled Baijiu, and pre-processing methods are used to suppress noise and enhance the image. Secondly, to segment the moving targets in the bottled Baijiu, the adaptive GMM fusion frame difference method is used, and then the minimum outer rectangle is carried out to crop the moving targets. Thirdly, the HOG features of the moving target in images are extracted. Finally, SVM is selected to detect both bubbles and impurities in bottled Baijiu.

In summary, this paper proposes a novel HOG-SVM impurity detection method for bottled Baijiu, a specific food product in China, based on adaptive GMM fusion frame difference. In this paper, not only the image difference mechanism between background and foreground pixels and the Gaussian mixture model learning rate is clarified, but also the Gaussian function merging mechanism and the coupling relationship between the frame difference algorithm and Gaussian mixture background subtraction method are revealed. HOG-SVM impurity detection method has overcome the bottleneck problems of the slow speed and low efficiency of the traditional manual lamp inspection. This study provides a new non-destructive inspection method to avoid impurities in Baijiu products from damaging consumer health and can improve food safety.

## 2. Materials and Methods

### 2.1. Image Data Acquisition of Impurities

#### 2.1.1. Image Acquisition System

**Figure 1:**
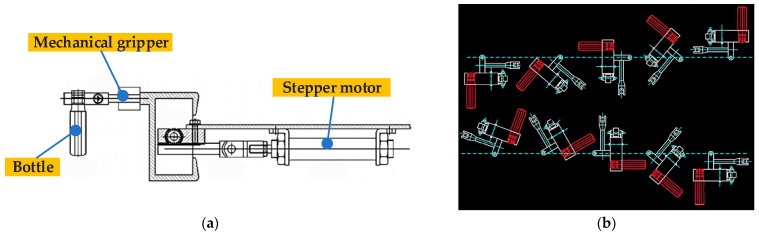
Diagrams of the flipping mechanism and the flipping process of a bottled Baijiu. (**a**) The flipping device; (**b**) The flipping process of bottled Baijiu.

When detecting impurities in Baijiu, stains and scratches on the bottle surface are easily misjudged as impurities of Baijiu liquid, so a bottle flipping mechanism is introduced into the image acquisition system in this study. As the impurities will move when flipping the bottle, the static bottle stains and scratches can be easily distinguished. The structure of the flipping device is shown in Figure 1a, and the process of flipping the bottle is shown in Figure 1b.

**Figure 2:**
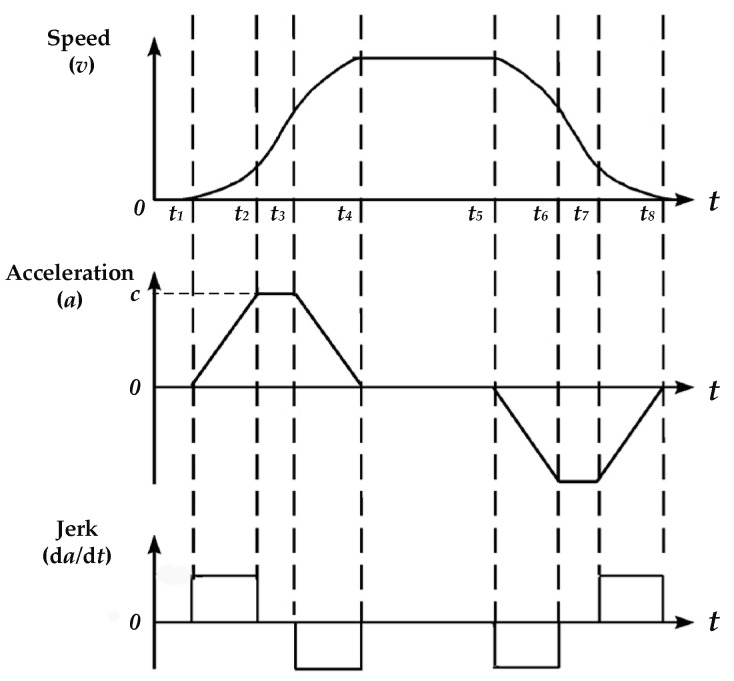
The timing diagram of the stepper motor used in the flipping device.

As shown in Figure 1b, the bottled Baijiu needs to be turned twice to meet the requirements of detecting the white and black impurities. In the flipping process, if the flipping force is too strong or the flipping device moves too quickly, there will be a large number of air bubbles in the bottle. So, an S-type stepper motor speed control algorithm [26] is used to make it gentler when flipping the bottle. In this way, the flipping process is divided into seven stages. As shown in Figure 2, the acceleration of the flipping device increases gradually from zero during the period *t*_1_~*t*_2_ and keeps the same during the period *t*_2_~*t*_3_. The acceleration decreases gradually from the max value *c* to 0 during the period *t*_3_~*t*_4_. Further, the period *t*_4_~*t*_5_ is a smooth operation stage during which the rotation speed of the flipping device stays unchanged, i.e., the acceleration is zero. The period *t*_5_~*t*_8_ is the deceleration stage and is just the opposite of the process in the acceleration stage, during which the rotation speed decreases gradually from the maximum to zero with a negative acceleration. During the whole period, the absolute value of jerk is unchanged.

**Figure 3:**
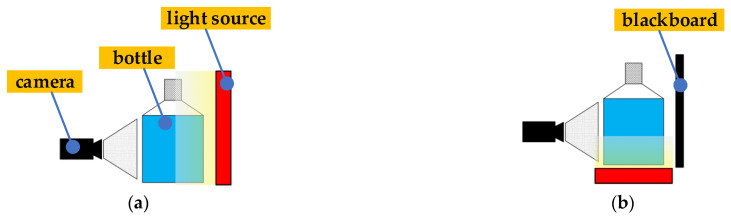
Black and white impurity lighting schemes. (**a**) Black impurity lighting scheme; (**b**) White impurity lighting scheme.

The red LED with a parallel light plane is chosen as the light source because the area of the impurities in Baijiu is usually tiny, and the wavelength of this red LED light is closer to the peak sensitivity of the sensor used in the image acquisition system. So, it is easier for the industrial camera to capture the tiny impurities in Baijiu with such an LED. Baijiu impurities are divided into two kinds by colour, black and white. Additionally, two lighting schemes have been developed as shown in Figure 3.

**Figure 4:**
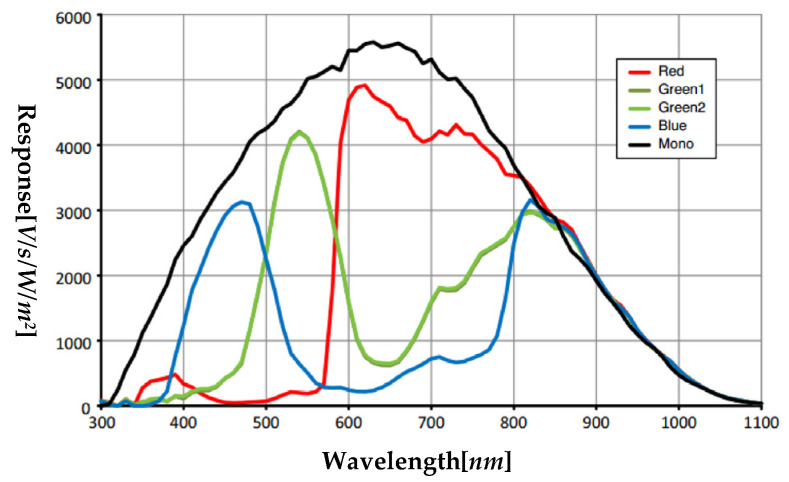
The response curve of the industrial camera sensor.

During the image acquisition process, the parameters of the camera directly determine the quality of images and are very crucial for the subsequent analysis of those images. In this paper, the MV-CA050-20GM/GC industrial area scan camera produced by HIKVISION (Hangzhou, China) is selected. The sensor response curve of the camera is shown in Figure 4, and both the main performance parameters and lens parameters of the camera are shown in Table 1.

#### 2.1.2. Image Preprocessing

**Figure 5:**
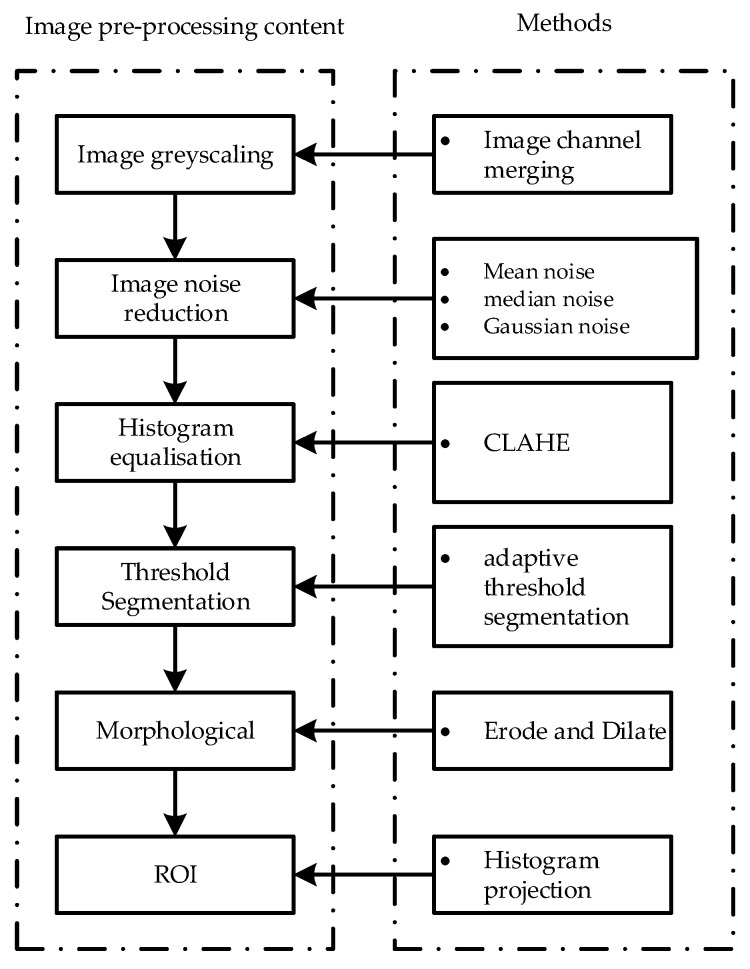
Preprocessing steps for the original images.

During the image acquisition process, as there are noise interferences and uneven illumination, the impurity features in the original image are not prominent. In addition, the original image is so informative that there are lots of redundant data and non-detected regions in it, which affects the real-time performance of the detection algorithm. Therefore, this paper performs noise reduction, threshold segmentation, histogram equalisation, and morphological operation on the original image to achieve image noise suppression and enhance the image quality, which provides high confidence input data for the subsequent detection algorithm. The image preprocessing steps in this paper are shown in Figure 5.

### 2.2. Detection and Identification of Impurities in Bottled Baijiu

#### 2.2.1. Adaptive GMM Fusion Frame Difference Algorithm

Different types of impurities move at different speeds. The background subtraction method is more sensitive to low-speed impurities, while the inter-frame differencing method is robust to high-speed impurities. In this paper, the two detection algorithms are combined and applied to detect impurities in bottled Baijiu to obtain optimal detection results.

##### GMM Model

The Gaussian mixture model is an online learning mixture background model [27,28], consisting of 3~5 Gaussian distributions [29]. The probability of occurrence of a certain pixel value in an image is obtained by weighting the probability and modelling its background. The probability density function $p\left(X_{t}\right)$ is calculated as follows:

(1) $$p\left(X_{t}\right)=\sum_{i=1}^{K}w_{i,t}p_{i}(x)=\sum_{i=1}^{K}w_{i,t}\eta \left(X_{t},\mu_{i,t},\sum i,t\right)$$

where $X_{t}$ is the pixel value at time *t*, $w_{i,t}$ is the weight, $\mu_{i,t}$ is the mean vector, and ∑i,t is the covariance matrix [30]. The density function of the *i*_th_ Gaussian distribution at time *t* is calculated as follows:

(2) $$\begin{array}{c} \eta \left(X_{t},\mu_{i,t},\sum i,t\right)=\\ \frac{1}{\sqrt{{\left(2\pi \right)}^{n}\left|\sum i,t\right|}}e^{-\frac{1}{2}\left(X_{t}-\mu_{i,t}\right)T_{\Sigma }{-}_{i,t}^{-1}\left(X_{t}-\mu_{i,t}\right)} \end{array}$$

Assuming that each pixel has *K* Gaussian distributions, the following conditions need to be met compared to the pixel values *X_t_*.

(3) $$\left|X_{t}-\mu_{i,t-1}\right|⩽D\times \delta_{i}$$

If Equation (3) is satisfied, then the related pixel is matched to the current Gaussian distribution. If Equation (3) is not satisfied, the Gaussian distribution with the lowest priority is replaced with a new Gaussian distribution, and the weight values of each Gaussian distribution are then normalised. According to $w_{i,t-1}/\delta_{i,t-1}$, and $\delta_{i,t}$, the priorities are ranked usually by taking the larger value.

In the parameter update stage, the weights of all the Gaussian distributions are updated as in Equation (4), no matter if Equation (3) is satisfied or not. However, only if those distributions satisfy the Equation (3), their mean vectors and covariance matrices are updated according to the Equations (5) and (6).

(4) $$w_{i,t}=(1-\alpha )w_{i,t-1}+\alpha M_{i,t}$$

(5) $$\begin{array}{l} \mu_{i,t}=(1-\rho )\mu_{i,t-1}+\rho X_{i,t}\\ \rho =\alpha \eta \left(X_{t},\mu_{i,t},\delta_{i,t}\right) \end{array}$$

(6) $$\delta_{i,t}^{2}=(1-\rho )\delta_{i,t-1}^{2}+\rho {\left(X_{i,t}-\mu_{i,t}\right)}^{T}\left(X_{i,t}-\mu_{i,t}\right)$$

where *α* is the learning rate, *ρ* is the parameter update rate. If the related pixel does not match the current Gaussian distribution, then $M_{i,t}=0$, otherwise $M_{i,t}=1$ [31].

GMM models with high weights have the same high probability of describing the image background, so a fixed number of distributions is chosen as follows:

(7) $$B={\mathrm{argmin}}_{b}\left(\sum_{i=1}^{b}w_{i,t}>T\right)$$

where *B* is the number of Gaussian distributions, and *T* is the proportion of the background.

##### Adaptive GMM Model

In the actual production line, the traditional GMM background subtraction method has the following problems:

(1) The number of Gaussian distributions is fixed. So, lots of useless distributions are included, resulting in a large amount of computation and poor real-time detection performance.
(2) The model learning rate is fixed, which leads to poor robustness of the background area and ghosting images in the foreground area.
(3) It is susceptible to light changes and prone to produce false targets for suspended impurities.

**Figure 6:**
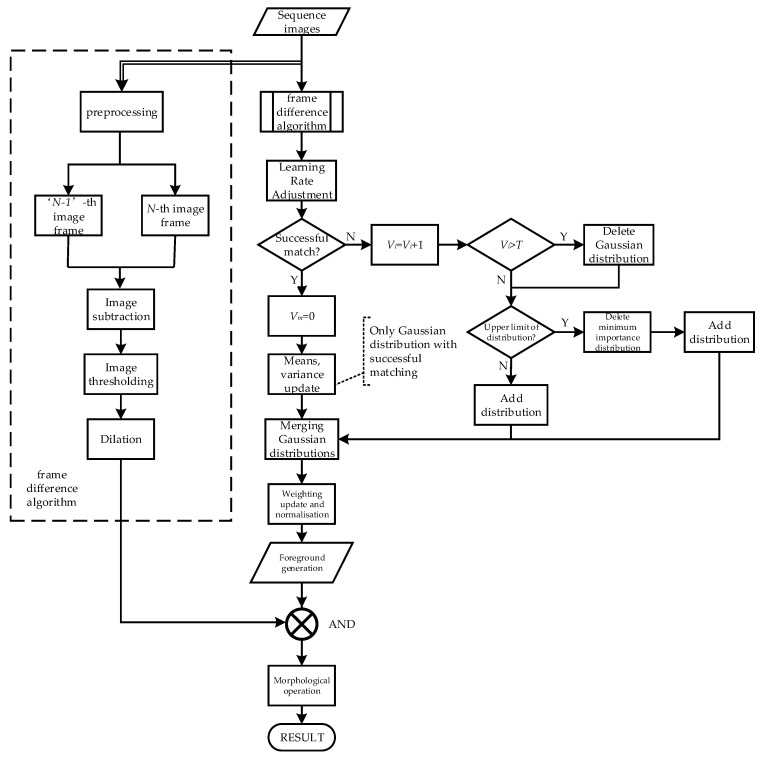
The flow diagram of the detection algorithm.

It is found that the background of the lamp inspection in the Baijiu factories is fixed when detecting impurities. So, to solve these problems mentioned above, some steps can be taken to counteract the effects of ambient light changes. As shown in Figure 6, in this paper, the Gaussian distributions of unmatched consecutive frames are appropriately removed in the GMM background modelling process so that fewer distributions are used to represent the observed values. Then the frame difference method is fused to eliminate the influence of ambient light changes. In Figure 6, *V_i_* is the continuous unmatched cumulative variable of the *i*_th_ distribution, and i∈(1,2,⋯,K), *V_m_* is the constant unmatched cumulative variable of the matching distribution and will be set to zero when the matching is successful.

The steps of the impurity detection algorithm based on the adaptive GMM fusion frame difference are as follows:

(1) For the initial observation *X_t_*, its mean *μ*_0_ and variance $\sigma_{0}^{2}$ are initialized as follows:

(8) $$\mu_{0}=\frac{1}{N}\sum_{t=1}^{N}X_{t}$$

(9) $$\sigma_{0}^{2}=\frac{1}{N}\sum_{t=1}^{N}{\left(X_{t}-\mu_{0}\right)}^{2}$$

where *N* is the number of frames, and *X_t_* is the pixel value at time *t*.

(2) It is assumed that frames $f_{t-1}(i,j)$ and $f_{t}(i,j)$ are the two consecutive frames of the sequence images at the coordinates (*i*, *j*), respectively. Firstly, the image difference is calculated between the two frames, and then the maximum difference result is used to obtain the frame difference result of the motion target. The corresponding calculations are listed as follows:

(10) $$D_{t}(i,j)=|f_{t}(i,j)-f_{t-1}(i,j)|$$

(11) $$f_{t}(i,j)=\{\begin{array}{ll} B_{t}(i,j), & D_{t}(i,j)\leq T\\ M_{t}(i,j), & D_{t}(i,j)>T \end{array}$$

(12) $$\{\begin{array}{l} T=T_{c}+T_{r}\\ T_{r}=\frac{1}{N}\sum \left|f_{t}(i,j)-f_{t-1}(i,j)\right| \end{array}$$

where $D_{t}(i,j)$ is the result of inter-frame differencing, $B_{t}(i,j)$ is the background area, $M_{t}(i,j)$ is the moving target, *T_c_* is a fixed value, and *T_r_* is the ambient complementary factor which is determined by ambient light changes.

According to the frame difference results, the motion target and background regions can be quickly divided. The initial value of the motion target pixel learning rate is greater than that of the background pixel. Further, a variable *G* (0≤G≤m) is created as the pixel model learning rate coefficient and is initialised to 0.5*m* (*m* is the upper limit of *G*). The relationship between the dynamic value of *G* and the learning rate *α* is listed as follows:

(13) $$G=\{\begin{array}{ll} G-1/\beta & f_{t}(i,j)\in B_{t}(i,j)\\ G+1 & f_{t}(i,j)\in M_{t}(i,j) \end{array}$$

(14) $$\alpha =\frac{2G}{m}\times \alpha \;0\leq C\leq m$$

where *β* is determined by the external environment and camera parameters.

(3) According to Equation (3), it is determined whether the observed pixel $X_{t}$ matches the Gaussian distribution. The continuous unmatched cumulative variable $V_{i}$ i∈(1,2,⋯,K) is introduced to remove the redundant Gaussian distribution. The parameters of the matched Gaussian distribution are updated according to Equations (5) and (6), and the parameters of the unmatched Gaussian distribution remain unchanged. If all Gaussian distributions are not successfully matched, the least significant Gaussian distribution needs to be replaced. To achieve a dynamic increase in the number of Gaussian distributions, the newly added Gaussian distribution parameters are updated as follows:

(15) $$\omega_{k+1,t}=\omega_{k,t}(1-\beta ),\beta \in \left(0,1\right)$$

(16) $$\mu_{k+1,t}=X_{t}$$

(17) $$\sigma_{k+1,t}^{2}=(1+\beta )\times \sigma_{k,t}^{2}$$

where *β* is a factor to maintain the weight and variance of the newly added Gaussian distribution at an appropriate level.

When the difference between the means of the two Gaussian distributions is within a specified range, they will be merged. The parameters of the distribution after being merged are set as follows:

(18) $$\omega_{c,t}=\omega_{a,t}+\omega_{b,t}$$

(19) $$\mu_{c,t}=\frac{\omega_{a,t}\times \mu_{a,t}+\omega_{b,t}\times \mu_{b,t}}{\omega_{a,t}+\omega_{b,t}}$$

(20) $$\sigma_{c,t}^{2}=\frac{\omega_{a,t}\times \sigma_{a,t}^{2}+\omega_{b,t}\times \sigma_{b,t}^{2}}{\omega_{a,t}+\omega_{b,t}}$$

where $\omega_{c,t}$ is the weight of the combined distribution, $\mu_{c,t}$ is the mean of the combined distribution, and $\sigma_{c,t}^{2}$ is the variance of the combined distribution.

(4) After the matching comparison is completed, all Gaussian distributions’ weights are updated according to Equation (4) and are normalised according to the following equation:

(21) $$\omega_{k}=\omega_{k}/\sum_{i=1}^{N_{u}}\omega_{i},k=1,2,\cdots ,N_{u}$$

(5) The result of the frame difference calculation and the adaptive GMM foreground image are subjected to a morphological process operation after a logic AND operation. According to the result from the above steps, the motion target is captured with a minimum outer rectangle.

#### 2.2.2. Impurities and Bubbles Differentiation Algorithm

**Figure 7:**
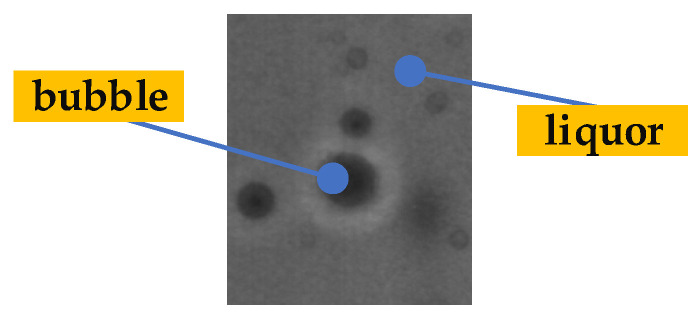
Air bubbles in Baijiu.

As shown in Figure 7, in the process of transferring and turning the bottled Baijiu, bubbles will inevitably be generated. There is no obvious difference in the motion characteristics between bubbles and impurities. So, bubbles are easily misjudged as impurities.

**Figure 8:**
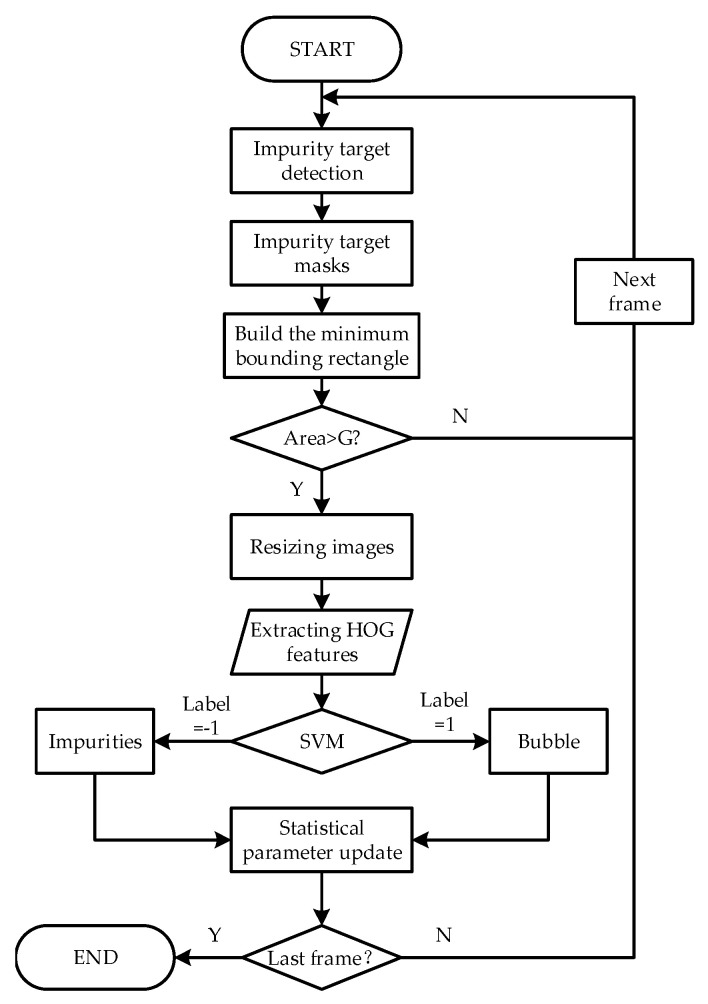
Flow diagram of HOG-SVM recognition algorithm.

At present, big data and neural networks [32] are commonly used to recognise images. However, the autonomous transformation of the Baijiu industry in China is relatively late, and there is a lack of image data accumulation. Additionally, because the bubbles are either elliptical or circular, the edge grey value of the bubbles changes significantly. So, a non-sliding window bubble recognition algorithm based on HOG-SVM is designed, as shown in Figure 8.

**Figure 9:**
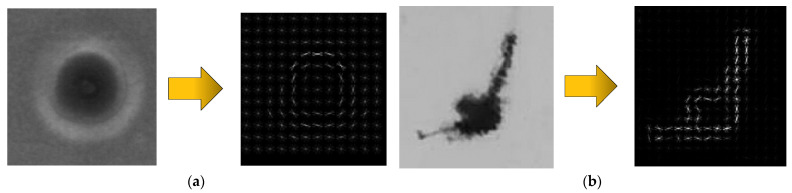
Examples of HOG characteristics of a bubble and an impurity. (**a**) Bubble HOG feature extraction result; (**b**) Impurity HOG feature extraction result.

As shown in Figure 8, firstly, the aforementioned moving target (i.e., the impurity) detection algorithm is used to obtain the rectangular frame mask in the HOG-SVM algorithm. Then the HOG features of the image within the rectangular frame are extracted. Compared with the traditional multi-scale sliding window retrieval method, this method can reduce the amount of computation. To further improve the real-time performance of the system, this study votes for the gradient direction in each cell through linear interpolation. Further, blocks of various scales are used to complete feature structure adjustment and feature selection to finally form a multi-scale block. An example of the HOG feature of the bubble and impurity is shown in Figure 9.

The previously extracted HOG feature data of bubbles and impurities are fed into the SVM model, and the radial basis kernel function is chosen for the SVM model [33,34]. The radial basis kernel function is calculated as follows:

(22) $$k\left(x,x^{\prime }\right)=\exp\left(-\frac{{\left\|x-x^{\prime }\right\|}^{2}}{2\sigma^{2}}\right)$$

where ${\left\|x-x^{\prime }\right\|}^{2}$ is the squared Euclidean distance between the two eigenvectors of impurities and bubbles, and *σ* is the free parameter.

**Figure 10:**
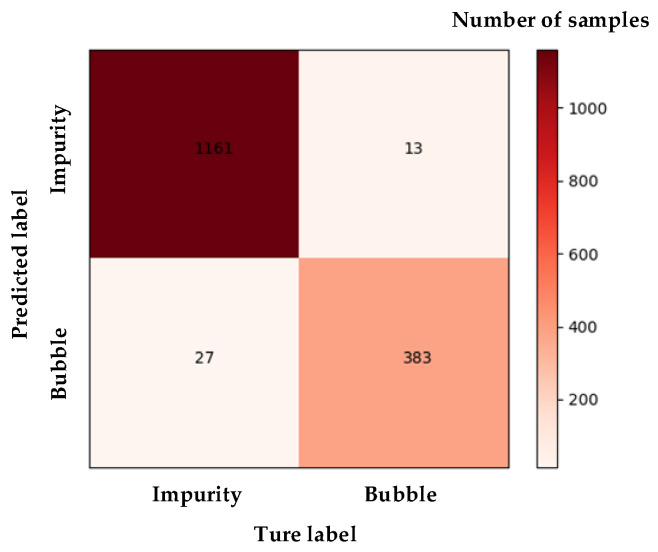
Confusion matrix of impurity and bubble identification results.

According to the sample ratio of 3:1, 1188 impurity samples and 396 bubble samples were selected. The samples were input into the HOG-SVM model to obtain the identification results of impurities and bubbles. The confusion matrix of the HOG-SVM model is given in Figure 10.

As shown in Figure 10, the grid on the diagonal of the confusion matrix is darker, with 97.7% and 96.7% accuracy for the within-class recognition, respectively.

## 3. Experiments and Results

**Figure 11:**
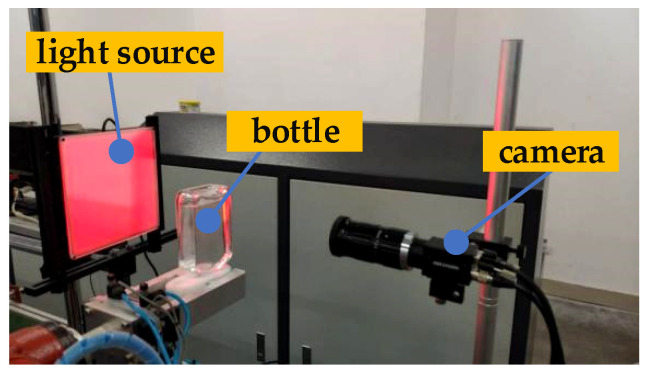
The position relationship between the light source, the camera and the bottle.

The experiments in this paper were carried out on a 64-bit Lenovo (Beijing, China) PC with a Intel (Santa Clara, CA, United States) CPU of i7-11800H and a Nvidia (Santa Clara, CA, United States) GPU of RTX3070. The centre of the planar light source (i.e., the aforementioned red LED), the industrial camera and the bottled Baijiu to be inspected are all located on the same level, as shown in Figure 11.

**Figure 12:**
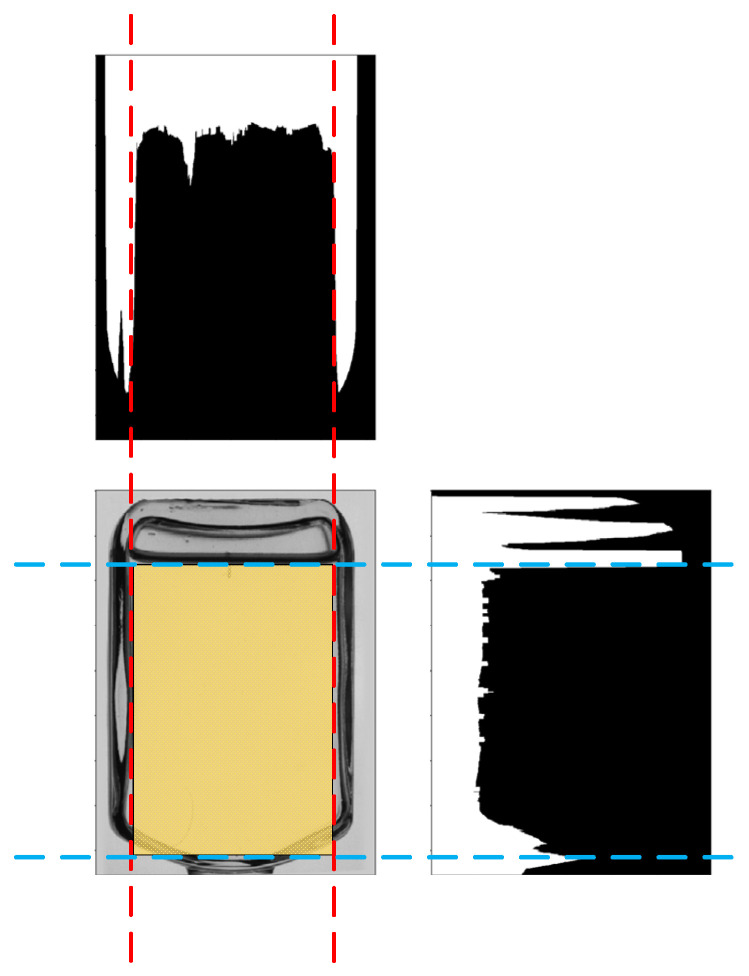
Calibration of the impurity detection area for bottled Baijiu.

To avoid inputting useless information to the detection system, such as bottle walls, background instruments and caps, a histogram projection of the binary image was calculated. According to the projection result, the image ROI was cropped by positioning the histogram peaks. The process is shown in Figure 12 where the yellow part of the figure is the impurity detection region.

### 3.1. Comparative Experiments of Detection Algorithms

#### 3.1.1. Comparison with Image Processing Based Detection Algorithms

**Figure 13:**
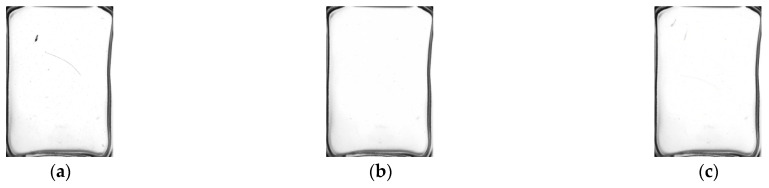
Comparison of background modelling results. (**a**) Original image; (**b**) Background model of this paper; (**c**) Background model of the literature [35].

**Figure 14:**
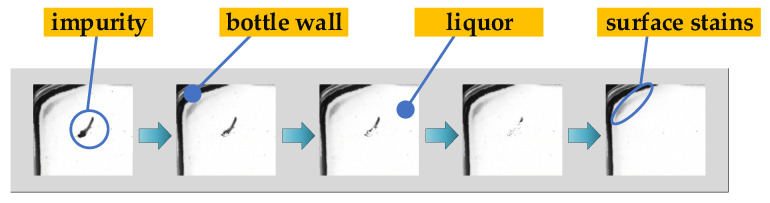
Background convergence process of the adaptive GMM fusion frame difference.

The background modelling effect comparison experiment was carried out between the algorithm in this paper and the algorithm in the literature [35]. The results are shown in Figure 13. It can be seen from the figure that there is an afterimage in the background model of the algorithm in the literature [35], indicating that the background learning rate of the algorithm in the literature [35] is not properly selected. The adaptive GMM fusion frame difference method is better in background modelling and can quickly converge the impurity historical pixel points to the background. The convergence process is shown in Figure 14.

**Figure 15:**
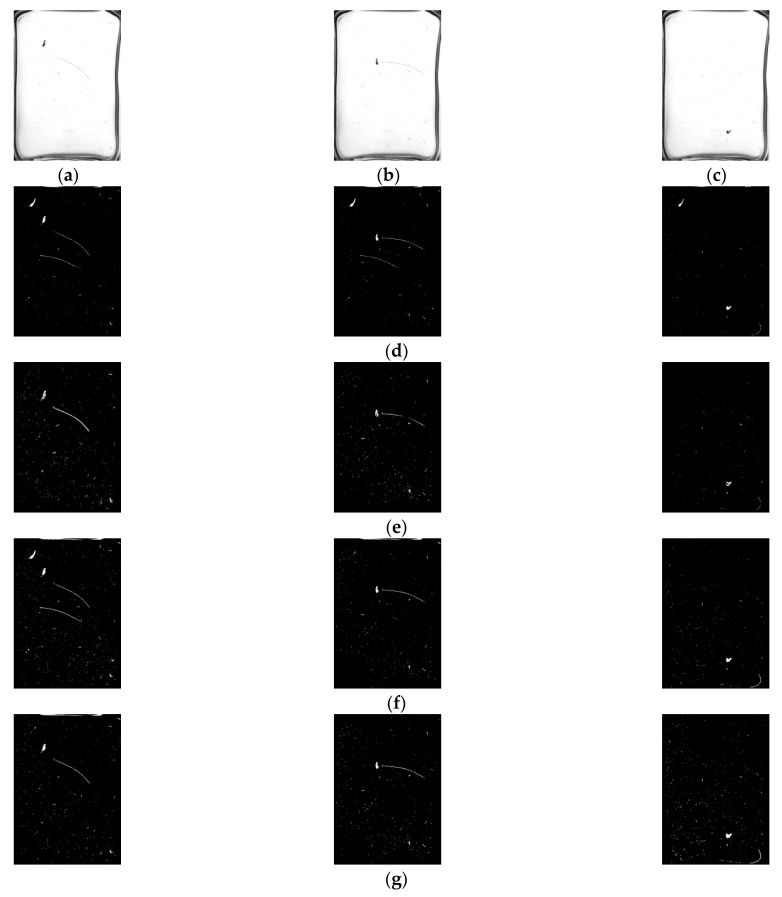
Examples of different algorithms for detecting impurities in bottled Baijiu. (**a**) Frame 34; (**b**) Frame 63; (**c**) Frame 212; (**d**) Result of the algorithm in the literature [36]; (**e**) Result of the frame difference method; (**f**) Result of traditional GMM background differential detection method; (**g**) Result of the algorithm in this paper.

The experimental video was divided into three frame segments: start, run and end. A set of frames would be randomly selected from the three frame segments during the experiment. In this study, frames 34, 63 and 212 were chosen. The algorithm in this paper was compared with the traditional GMM background subtraction method, the frame difference method and the method in the literature [36]. The comparison results are shown in Figure 15.

**Figure 16:**
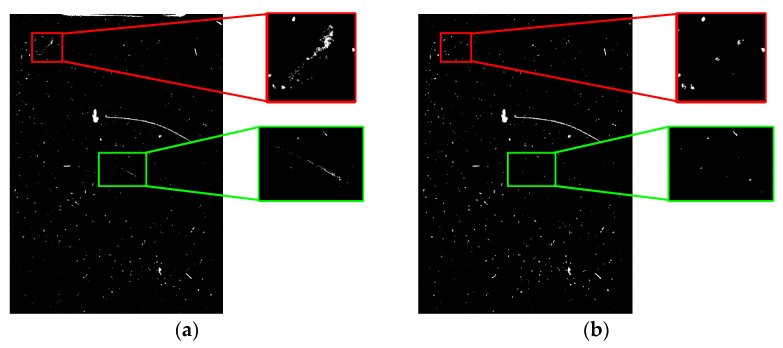
A partially enlarged display of the test results. (**a**) Traditional GMM detection result of frame 63; (**b**) Detection result of frame 63 based on the algorithm in this paper.

As shown in Figure 15, the algorithm of the literature [36] is more prone to give false detection results for impurities throughout the detection process, especially for impurities with larger areas, and is too slow to update the background. The frame difference method is accurate in detecting impurities, but there is lots of noise in the foreground. Further, it is not effective in detecting long, thin impurities, such as hair. The traditional GMM background subtraction method shows false targets in the results of frame 34. For the convenience of observation, the results of the 63rd frame detected by the traditional GMM and the algorithm in this paper are partially enlarged and displayed, as shown in Figure 16. It can be seen that the algorithm proposed in this paper can quickly achieve the foreground mask generation of the moving target by appropriately selecting the background learning rate.

According to Equations (23)–(25), the precision rate *P_a_*, the recall rate *P_b_*, the comprehensive index *P_c_* and the FPS (frames per second) of each detection algorithm are calculated as follows:

(23) $$P_{a}=\frac{M}{M+N}$$

(24) $$P_{b}=\frac{M}{M+L}$$

(25) $$P_{c}=2\times \frac{P_{a}\times P_{b}}{P_{a}+P_{b}}$$

where *M* is the number of correctly detected foreground pixels, *N* is the number of background pixels incorrectly detected as foreground pixels, *L* is the number of foreground pixels incorrectly detected as background pixels, and *P_c_* is a comprehensive indicator.

**Figure 17:**
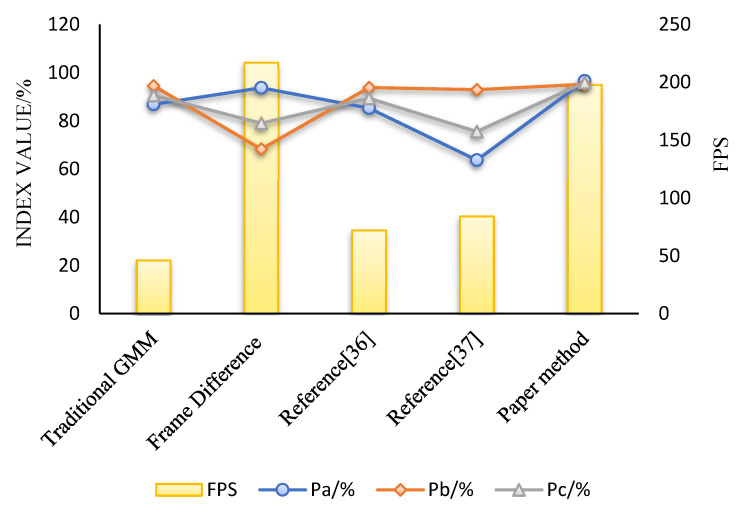
Distribution diagram of evaluation indicators for each algorithm.

The experiment results are shown in Table 2 and Figure 17.

It can be seen from Table 2 that the precision and recall rates of the algorithm in this paper are 96.6% and 95.2%, respectively, and its comprehensive index is up to 95%. So, the algorithm in this paper can accurately detect moving impurities and has a lower error rate. As known in Figure 17, the FPS of the algorithm in this paper is slightly lower than that of the frame difference method, but it can also meet the needs of real-time detection. In addition, the three indicators of *P_a_*, *P_b_*, and *P_c_* of the algorithm in this paper are better than other algorithms. So, our method has strong comprehensive performance and is more suitable for impurity detection of bottled Baijiu.

#### 3.1.2. Comparison with Machine Learning-Based Detection Algorithms

**Figure 18:**
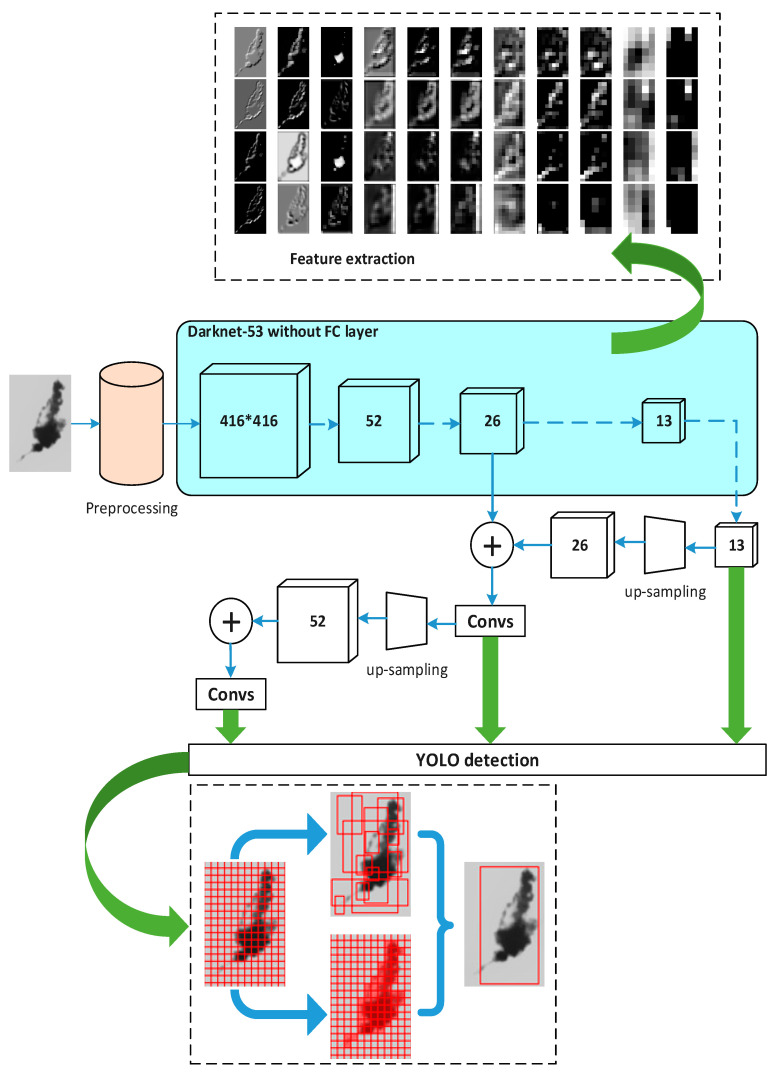
Schematic diagram of YOLOv3 network structure for impurity detection.

KNN (K-Nearest-Neighbor) [37,38,39] and YOLOv3 (You Only Look Once Version 3) [40,41] are two kinds of commonly used machine learning methods to detect impurities in Baijiu, as YOLOv3 has good performance in target detection and has become one of the current research hotspots. This paper describes YOLOv3 in detail and compares it with our method. The YOLOv3 model constructed in this paper uses Darknet-53 as the feature extraction layer. The 74-feature extraction layers consist of a large number of residual networks. By fusing the upsampled feature maps rich in high-level semantic information with low-level feature maps rich in location information, the prediction of detection boxes and categories is performed on three scale feature images downsampled by 32, 16, and 8 times, respectively. The YOLOv3 network structure used in this experiment for comparison is shown in Figure 18.

Five experiments were performed, and in each time 1000 pictures were tested. The pictures with impurities and those without impurities accounted for 50% each, and the average recognition rate was calculated as follows:

(26) $$\eta =\frac{n_{1}+n_{2}}{m\times n_{3}}\times 100\%$$

where *m* is the number of experiments, *n*_1_ is the number of times that an experimental sample containing impurities is correctly detected, *n*_2_ is the number of times that an experimental sample containing no impurities is correctly detected, *n*_3_ is the total number of detections per round, and *η* is the recognition rate.

The experimental results of comparing our detection algorithm with KNN and YOLOv3 are shown in Table 3.

It can be seen from Table 3 that the recognition rate of our algorithm is similar to the two machine learning algorithms, i.e., KNN and YOLOv3. However, the machine learning algorithms contain complex network structures, a large number of training samples is required in the early stage, and expensive hardware equipment is needed to run such complicated algorithms. However, the algorithm in this paper can not only run smoothly on a low-cost hardware platform, but also only requires a small number of samples, and the recognition performance is close to that of the machine learning algorithm, or even better.

### 3.2. Comparison Experiment of Illumination Change

**Figure 19:**
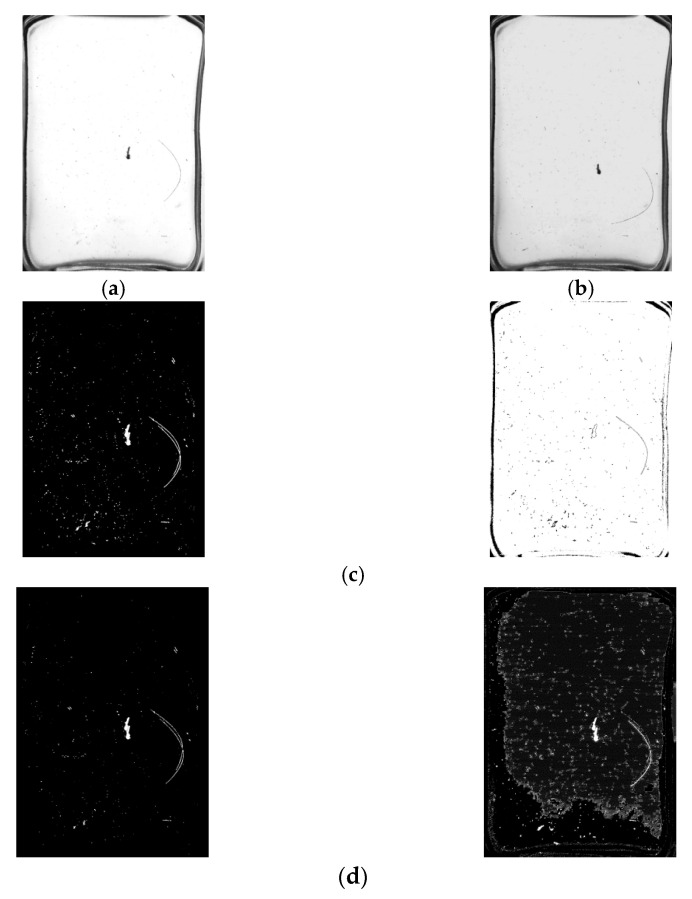

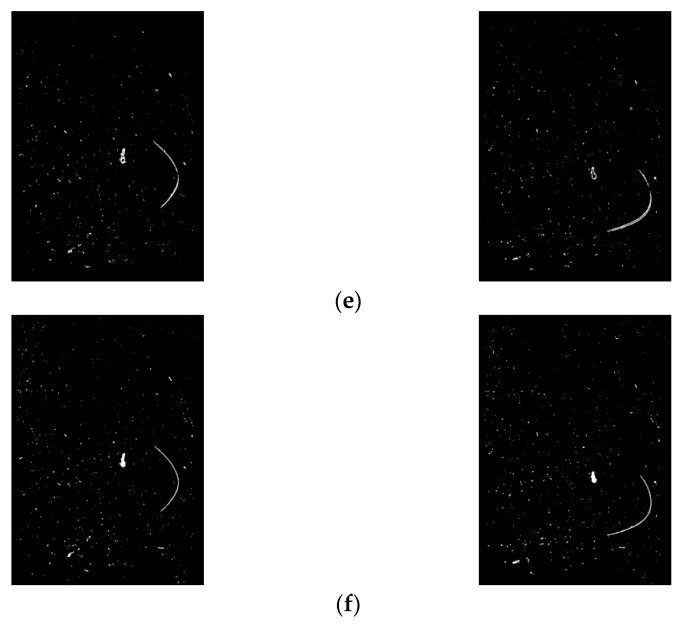
Examples of detection results for different algorithms when the light changes. (**a**) Frame 124; (**b**) Frame 144; (**c**) Result of traditional GMM background differencing method; (**d**) Result of the algorithm in literature [36]; (**e**) Result of frame difference method; (**f**) Result of method in this paper.

Scene illumination changes generally exist in the process of impurity detection of bottled Baijiu. Therefore, the 124th and 144th frames were selected in this experiment because the experimental video shows a change from light to dark in the 124th to 144th frames, as shown in Figure 19. It can be seen that the traditional GMM algorithm is less robust to light changes, and the algorithm of literature [36] is also more sensitive to light changes. The frame difference method has good resistance to light changes, but the Hole phenomenon is apparent. In this paper, the adaptive GMM algorithm is fused with the frame difference method, which not only avoids the Hole phenomenon but also eliminates the interference of light change.

### 3.3. Comparison Experiments with Manual Lamp Inspection

At present, the manual lamp test method is widely used in the Baijiu factory to detect impurities in bottled productions. So, it is of practical importance to conduct comparative experiments between the method of this paper and the manual lamp test. Comparison experiments regarding detection accuracy have been performed in this study for different impurity detection performance, repeatability index, Knapp-Kushner test and detection speed.

#### 3.3.1. Comparison of Detection Performance

**Figure 20:**
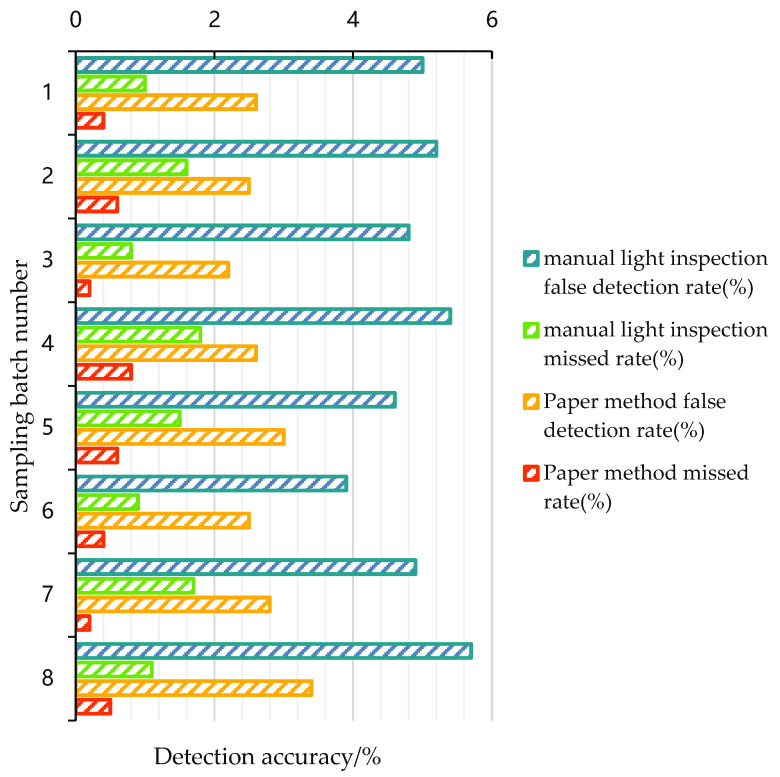
Accuracy results of the algorithm in this paper and manual lamp inspection.

Two specifications products of 40° and 52° Baijiu were selected as test objects, and four batches of each specification of Baijiu products were taken. In total, 500 bottles were taken from each batch and tested at a rate of 800 bottles per hour. The experiment was carried out according to the distillery standards, and the false detection rate and missed rate were counted. The experimental results are shown in Figure 20. Our method had a missed rate of 1% and a false detection rate of around 3%, i.e., our algorithm can identify impurities that the human eye cannot detect.

#### 3.3.2. Comparison of Different Impurity Detection

The false detection rate and missed detection rate under different detection methods are counted for different types of impurities. In the real production process, glass shavings, mosquitoes and hair impurities are likely to appear in bottled Baijiu. Therefore, during this experiment, 100 bottles of black impurities and 100 bottles of white impurity test objects were prepared according to the probability of occurrence of various impurities. The black samples contained 40 bottles of mosquitoes, 40 bottles of hair, and 20 bottles of other black slags (mainly rust blocks, stone particles, etc.). The white ones contained 80 bottles of glass chips and 20 bottles of white fibres. To enrich the dataset, another 200 bottles of qualified samples were prepared as well. The detection results of the samples by our method and the manual light inspection method are shown in Table 4.

It can be seen from Table 4 that the average false detection rate and average missing detection rate of our method are 2.7% and 0.7% which are 2.8% and 0.9% higher than that of manual lamp inspection, respectively. It is verified that the detection performance of our method for various types of impurities is an improved method, compared with that of the manual lamp inspection.

#### 3.3.3. Comparison of Repeatability Indicators

Repeatability is one of the most critical performance indicators for Baijiu companies, and repeatability experiments are used to verify the consistency of results after the same batch of products has been tested several times. In this paper, 200 bottles of liquor samples were selected and marked with numbers 1 to 200 on the caps. The samples with numbers 9, 18, 57, and 81 contained impurities. The test was repeated 10 times by the manual lamp inspection method and the algorithm in this paper, respectively, and the number of inferior products obtained in each round was counted. The experimental result is shown in Table 5.

It can be seen from Table 5 that our method has detected defective products numbered 18, 57, and 86 in each round of the experiment, and the defective product numbered 9 has 1 missed inspection. By contrast, the manual lamp inspection method detected defective products numbered 57 and 86 successfully in each round of the experiment, but the defective products numbered 9 and 18 were missed twice and once, respectively. Moreover, the defective product numbered 40 was mistakenly detected once by the manual lamp inspection. The analysis results show that the repeatability index of our method is better than that of manual lamp inspection.

#### 3.3.4. Comparison of Detection Speed

In total, six workers were arranged to test 500 bottles of Baijiu in separate periods. The time consumed by our method to complete the same number of testing tasks and manual lamp inspection were both recorded, as shown in Table 6. The average time consumed by manual inspection of a bottle of Baijiu is about 3.2 s, while the average time consumed by our method is only about 0.5 s. The system in this paper saves about 2.7 s compared with manual inspection, indicating that our method is five times faster than the manual lamp inspection and meets the real-time requirement.

#### 3.3.5. Knapp–Kushner Test Experiment

The Knapp–Kushner test is introduced to further compare the manual lamp inspection with our method. This test is recognised by the European Pharmacopoeia and the US FDA (U.S. Food Drug Administration) and is often used to evaluate the effectiveness of the lamp inspection process [42]. This paper selected 600 experimental samples, divided them into six batches, and randomly invited six workers to test each batch separately. Each batch consisted of 100 experimental samples, including 25 bottles of samples containing a large number of impurities, 25 bottles of samples containing a small number of impurities, and other randomly selected 50 bottles. Every bottle in each batch was numbered. Then, according to the Equations (27)–(30), the quality factor *FQ*, the manual inspection quality factor *FQA*, the machine inspection quality factor *FQB*, and the detection efficiency comparison value *R* of each sample were calculated as follows:

(27) $$FQ_{i}=\frac{q}{N}\times 10$$

(28) $$FQA_{\left(7,10\right)}=\sum_{i=1}^{M}FQA_{i}$$

(29) $$FQB_{\left(7,10\right)}=\sum_{i=1}^{M}FQB_{i}$$

(30) $$R=\frac{FQB_{\left(7,10\right)}}{FQA_{\left(7,10\right)}}\times 100\%$$

where *N* is the total number of tests, *q* is the number of rejections, *i* is the number of bottles with quality factors in the range of 7 to 10, and *R* is the test efficiency ratio.

**Figure 21:**
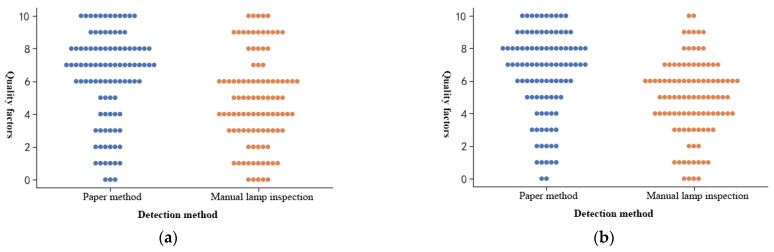

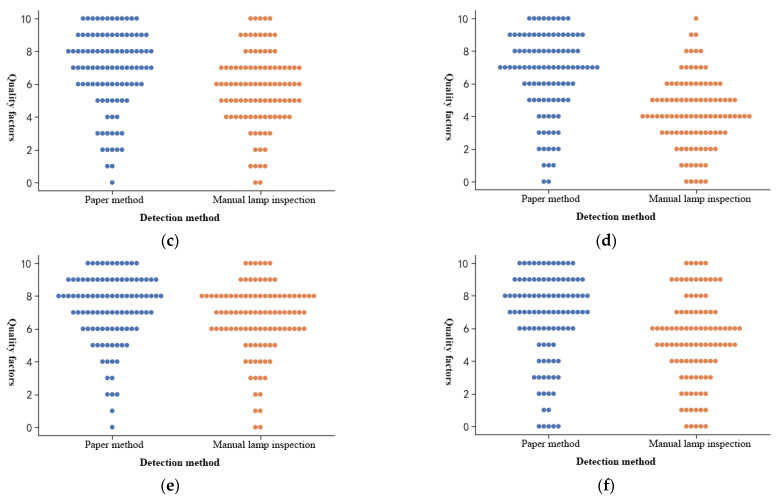
Statistical maps of the distribution of quality factors. (**a**) Experimental result of batch 1; (**b**) Experimental result of batch 2; (**c**) Experimental result of batch 3; (**d**) Experimental result of batch 4; (**e**) Experimental result of batch 5; (**f**) Experimental result of batch 6.

Statistical results of the quality factor distribution of the method in this paper and manual lamp inspection are shown in Figure 21.

As shown in Figure 21, the quality factors of the method in this paper are concentrated between 7 and 10 in each test, which is generally higher than that of the manual lamp inspection. In each batch, the detection efficiency ratio *R* of this paper’s method compared to manual lamp inspection meets R≥1, indicating that the overall detection performance of this paper’s method is better than that of manual lamp inspection.

## 4. Discussion

Due to food production processes and packaging technologies, impurities such as broken glass, hair and insect bodies may be present in bottled Baijiu, which may cause discomfort in the throat, oesophagus and stomach and even cause digestive disorders [43]. Further, some harmful substances may even be absorbed into the body and cause consumer poisoning [44]. Manual lamp inspection is commonly used in Chinese Baijiu processing plants to detect impurities in bottled products, and the quality of manual lamp inspection results varies from person to person. As it can be known in Figure 21 that the manual lamp inspection performs better than the method proposed in this paper when the quality factor for the first samples is 9. However, looking at all batch test results, it can be found that this situation just happens when the inspector is experienced with strong business skills. This situation is also found in batch 5, and the result is similar to the paper’s method when the inspectors are skilled. By contrast, samples in batch 4 are tested by a new inspector who is not skilled in the business, and the manual detection results are far from the results of our method. In addition, the manual lamp inspection detection rate decreases as the quality factor increases, indicating that manual lamp inspection is prone to fatigue after a lot of continuous labour, which affects detection efficiency. This can be confirmed in Table 5. Therefore, the results of manual lamp inspection are affected by various factors, such as inspectors’ personality, mood and physical ability, and are unstable. The proposed computer vision-based method for detecting impurities in bottled Baijiu is more stable and efficient than that. Figure 20 confirms that the average miss detection rate and the average false detection rate of this paper’s method are 45.3% and 64.4%, which are lower than manual lamp inspection, respectively. Table 6 shows that this paper’s method can perform detections more quickly.

The difficulty in detecting impurities in liquor based on machine vision technology lies in how to avoid misjudging scratches and stains on the surface of the bottle as impurities. Huang [7] detected impurities by rotating the bottle at a high speed to suspend them by centrifugal force. Li [12] fixed the bottle on a rotating wheel to suspend impurities by 180-degree inversion and detected them. This paper uses a similar idea of making the impurities move by flipping the bottle and then uses the method of detecting moving objects to obtain images of the impurities as well. Mahfouf [45] used the optical flow method to detect targets through transient changes generated by the movement of the targets, but the method was slow and not suitable for large batches of bottled Baijiu. Zhou [17,46] used the frame difference method to quickly detect moving impurities by choosing the time interval and threshold, which was computationally simple and could only extract the contours of the impurities. To obtain the complete impurity pixels, it is necessary to solve the Hole problem of the frame difference method. Yan [36] and Barnich [47] proposed the ViBe algorithm, a pixel-level target detection algorithm based on background model update, which could randomly update the background model. As the ViBe algorithm does not take into account the frame rate or colour space of the video, it cannot effectively suppress the residual shadow of the moving target, as shown in Figure 15b. However, the ViBe algorithm takes a long time to eliminate the residual shadow of the target and does not take into account changes in the background environment over time, which makes it insensitive to sudden changes in illumination. Moreover, it can be known from Figure 19c that the ViBe algorithm is unable to detect moving targets quickly when there are sudden changes in illumination. Additionally, when there are high-frequency disturbances in the background, the ViBe algorithm classifies the disturbances as impurity targets, resulting in false detection. In contrast, the traditional GMM algorithm proposed by Stauffer [48] is more robust than the ViBe algorithm because the algorithm uses a model that mixes multiple Gaussian distributions to model the sequence of pixels’ values for each pixel in the video frames. However, the number of Gaussian distributions and the learning rate of the traditional GMM algorithm are fixed, which will cause the algorithm to be computationally intensive and slow in background modelling. The adaptive GMM algorithm proposed in this paper can merge redundant Gaussian distributions and dynamically adjust the learning rate in combination with the frame difference method. As shown in Figure 15b,e, the background modelling speed of this paper’s method is faster. From Figure 16, it can be known that this paper’s method uses a more reasonable GMM learning rate, and from Figure 19, we can see that the paper’s method has better robustness.

It is also a difficult task to distinguish between impurities and bubbles in bottled Baijiu. Huang [49] used fuzzy least squares SVM to distinguish between small impurities and bubbles, which was similar to our method, but different classification features were used. Huang used greyscale, area, velocity and acceleration as classification features, which are not easy to obtain. However, this paper uses HOG features which are easier to calculate and are prone to obtaining a better result, as it can be known from Figure 10 that the entire sample identification accuracy of the method in this paper is up to 97.5%. Ge [50] used pulse-coupled neural networks (PCNN) to combine the continuity and smoothness of impurity trajectories to distinguish between impurities and bubbles. In addition, the popular YOLO neural network [40,41] is currently a hot research topic in the field of target detection. However, Chinese Baijiu is a special and very expensive foodstuff, and it is impossible to obtain enough images to train and test such a deep learning network. Analysis of Table 4 shows that the method in this paper is close to the neural network detection results, but the method in this paper only requires a small data set and does not require the construction of a complex neural network. Moreover, it has a short development cycle and does not need expensive high-performance equipment. Perhaps by continuing to increase the training samples, the neural network detection will give better results. However, as there is no available database of Chinese bottled Baijiu on the internet, it requires a lot of human and material costs to collect the data and lots of time to train the neural network itself. With the deepening of research and the increase of bottled Bajiu datasets, this research will explore a neural network in the future. Therefore, the method in this paper can be used as a proven and efficient tool for impurity detection in Chinese bottled Baijiu at present.

## 5. Conclusions

(1) In this paper, an image acquisition system was built, and a red planar LED with a wavelength closer to the peak sensitivity of the sensor was selected as the light source. Two illumination schemes have been developed to light from the bottom and back of the bottle, respectively. With the help of a flipping device which prompted impurities movement, high-quality images of target impurities were captured. The original images were then subjected to noise reduction, threshold segmentation, histogram equalisation and morphological operation. Further, the horizontal and vertical histogram projections of the original images were analysed to crop the ROI region for detection and to provide input data with distinct impurity features and low redundancy for later detection algorithm studies.
(2) An algorithm for Baijiu impurity detection based on an adaptive GMM fusion frame difference is proposed in this paper; this algorithm has fewer Gaussian distributions and can build a background model faster. The algorithm in this paper also shows better robustness when the light changes. Our method not only solves the Hole problem of the frame difference method but also can obtain the complete impurity pixels. Compared with the traditional GMM algorithm, the algorithm of this paper has reduced the number of redundant Gaussian distributions and can dynamically adjust the GMM learning rate. Based on the experimental analysis, it can be seen that the algorithm in this paper achieves an accuracy rate of 96.6% and a completeness rate of 95.2%. In addition, the repeatability index of the algorithm in this paper is better than the manual lamp inspection, with an average false detection rate and an average missed detection rate of 2.8% and 0.9%, respectively, and the detection efficiency ratio is greater or equal to 1, i.e., R≥1 , in the Knapp–Kushner test.
(3) To exclude bubble interference, HOG features were extracted from the segmented motion target images, and the SVM model was constructed using the radial basis kernel function. Validated on 1188 impurity samples and 396 bubble samples, the HOG-SVM algorithm achieved an accuracy rate of 97.5% for the entire sample, and the accuracy rates within the class are 97.7% and 96.7%, respectively.

In summary, the HOG-SVM impurity detection method based on adaptive GMM fusion frame difference has advantages in detection accuracy, repeatability index and detection speed compared to the current manual lamp inspection commonly used in the Baijiu factories. The research results in this paper can provide a specific reference for the construction of smart Baijiu factories in the future.

## Figures and Tables

**Table 1:** Performance parameters of the industrial camera.

Device	Parameter	Value
Industrial Camera	Pixel size	4.8 μm × 4.8 μm
	Resolution	2592 × 2048
	Sensor type	CMOS
	Sensor area (V × H × D/mm)	1″
	Exposure	59 μs~10 s
	Gain	0 dB~15 dB
	Communication interface	USB3.0
	Signal to noise ratio (SNR)	39.5 dB
Optical Lens	Focus	16 mm
	Aperture	F1.6~F16
	Working distance	0.1 m~∞
	Optical interface	C-Mount
	Angle of view	53.4° × 43.3° × 33.4°

**Table 2:** Comparison of detection performance based on image processing algorithms.

Algorithms	$P_{a}/\%$	$P_{b}/\%$	$P_{c}/\%$	FPS
Traditional GMM	86.9	94.5	90.5	46
Frame Difference Method	93.7	68.3	79.0	217
Literature [35]	85.3	93.8	89.3	72
Literature [36]	63.7	92.9	75.6	84
Method of this paper	96.6	95.2	95.9	198

**Table 3:** Experimental results of the algorithm in this paper, KNN and YOLOv3.

Algorithms	η/%	FPS
KNN	94.3	53
YOLOv3	96.0	157
Method of this paper	96.4	184

**Table 4:** Experiment results of different types of impurities.

Impurity Type		Method of This Paper		Manual Lamp Inspection	
		False Detection Rate (%)	Missing Detection Rate (%)	False Detection Rate (%)	Missing Detection Rate (%)
Black Impurities	Hairs	2.4	0.4	5.3	1
	Mosquitoes	2	0.3	4.7	0.5
	Black scum	2.6	0.8	5.8	2
White Impurities	Glass chips	3.4	0.9	6.2	2.1
	Fibres	3.1	1.2	5.5	2.8

**Table 5:** Statistical result of repeatability experiments.

Number of Experiments	Detection Method	
	Method of This Paper	Manual Lamp Inspection
1	9, 18, 57, 86	9, 18, 57, 86
2	9, 18, 57, 86	18, 57, 86
3	9, 18, 57, 86	18, 40, 57, 86
4	9, 18, 57, 86	9, 18, 57, 86
5	9, 18, 57, 86	57, 86
6	9, 18, 57, 86	9, 18, 57, 86
7	18, 57, 86	9, 18, 57, 86
8	9, 18, 57, 86	9, 18, 57, 86
9	9, 18, 57, 86	18, 57, 86
10	9, 18, 57, 86	9, 18, 57, 86

**Table 6:** Comparison table of the average time.

Group	Average Time Spent on Manual Inspection $t_{1}/s$	The Average Time Is Taken to Test This Paper $t_{2}/s$	$\Delta t\;=\;t_{1}\;-\;t_{2}/s$
1	2.5	0.5	2
2	3	0.6	2.4
3	3.5	0.4	3.1
4	4	0.6	3.4
5	2.8	0.3	2.5
6	3.3	0.5	2.8
Average	3.2	0.5	2.7

## Data Availability

The data that support the findings of this study are available on request from the corresponding author. The data are not publicly available due to privacy or ethical restrictions.

## References

[1] Jiang Y., Sun J., Yin Z., Li H., Sun X., Zheng F. (2020). Evaluation of antioxidant peptides generated from Jiuzao (residue after Baijiu distillation) protein hydrolysates and their effect of enhancing healthy value of Chinese Baijiu. J. Sci. Food Agric..

[2] Wu J., Huo J., Huang M., Zhao M., Luo X., Sun B. (2017). Structural Characterization of a Tetrapeptide from Sesame Flavor-Type Baijiu and Its Preventive Effects against AAPH-Induced Oxidative Stress in HepG2 Cells. J. Agric. Food Chem..

[3] Zheng X., Han B. (2016). Baijiu, Chinese liquor: History, classification and manufacture. J. Ethn. Foods.

[4] Liu H., Sun B. (2018). Effect of Fermentation Processing on the Flavor of Baijiu. J. Agric. Food Chem..

[5] Ye H., Wang J., Shi J., Du J., Zhou Y., Huang M., Sun B. (2021). Automatic and Intelligent Technologies of Solid-State Fermentation Process of Baijiu Production: Applications, Challenges, and Prospects. Foods.

[6] Zou W., Ye G., Zhang K. (2018). Diversity, function, and application of Clostridium in Chinese strong flavor baijiu ecosystem: A review. J. Food Sci..

[7] Huang B., Li J.F., Wang J.C., He Y.Z., Shang W. (2012). Impurity Detection Using Machine Vision. Adv. Mater. Res..

[8] Kato F., Miyakawa T., Shimizu I. Developmental research of visualization of spatial distribution and the behavior of particles in the room. Proceedings of the 20th Annual Tech. Meeting on Air Cleaning and Contamination Control.

[9] Shimizu I., Kato F., Ikeda K., Ohashi Y. (2004). A technique for making holograms easily and for measuring simultaneously the behaviour of particles of different sizes and/or shapes. Meas. Sci. Technol..

[10] Huang B., Wang P., Ma S.L. (2014). A Detection System of Impurity in Transparent Liquid. Adv. Mater. Res..

[11] Diaz C.A.R., Leal-Junior A., Marques C., Frizera A., Pontes M.J., Antunes P.F.C., Andre P.S.B., Ribeiro M.R.N. (2019). Optical Fiber Sensing for Sub-Millimeter Liquid-Level Monitoring: A Review. IEEE Sens. J..

[12] Li X., Qiao T., Pang Y., Zhang H., Yan G. (2018). A new machine vision real-time detection system for liquid impurities based on dynamic morphological characteristic analysis and machine learning. Measurement.

[13] Zhou B., Chen L., Wu L. An Intelligent Foreign Substance Inspection Method for Injection Based on Machine Vision. Proceedings of the the International Conference on Image, Vision and Intelligent Systems (ICIVIS 2021).

[14] Cano Marchal P., Martínez Gila D., Gámez García J., Gómez Ortega J. (2013). Expert system based on computer vision to estimate the content of impurities in olive oil samples. J. Food Eng..

[15] Huo Y., Lian Q., Yang S., Jiang J. (2021). A recurrent video quality enhancement framework with multi-granularity frame-fusion and frame difference based attention. Neurocomputing.

[16] Ju J., Xing J. (2019). Moving object detection based on smoothing three frame difference method fused with RPCA. Multimed. Tools Appl..

[17] Zhou K., Huang Y., Chen E., Yuan R., Zhang Z. (2020). Real-Time Detection and Spatial Segmentation of Difference Image Motion Changes. IEEE Access.

[18] Schmidt B.E., Sutton J.A. (2019). High-resolution velocimetry from tracer particle fields using a wavelet-based optical flow method. Exp. Fluids.

[19] Lee C., Lee T., Nonomura T., Asai K. (2020). Evaluating the applicability of a phase-averaged processing of skin-friction field measurement using an optical flow method. J. Vis.-Jpn..

[20] Song Z., Ali S., Bouguila N. (2020). Background subtraction using infinite asymmetric Gaussian mixture models with simultaneous feature selection. IET Image Process..

[21] Djerida A., Zhao Z., Zhao J. (2020). Background subtraction in dynamic scenes using the dynamic principal component analysis. IET Image Process.

[22] Kushwaha A., Khare A., Prakash O., Khare M. (2020). Dense optical flow based background subtraction technique for object segmentation in moving camera environment. IET Image Process.

[23] Kanagamalliga S., Vasuki S. (2018). Contour-based object tracking in video scenes through optical flow and gabor features. Optik.

[24] Shu J., Gao L.H. (2020). Improved TLD tracking algorithm using frame difference method and correlation filter. Comput. Eng. Des..

[25] Tang Y.Y., Yan Y.Y., Liu Y.A. (2012). Rapid flame detection with the application of GMM. Comput. Sci..

[26] Sun S.M., Wang J.H. (2015). Implementation of Stepper Motor’s S-Curve Trajectories Control. Appl. Mech. Mater..

[27] Greggio N., Bernardino A., Laschi C., Dario P., Santos-Victor J. (2011). Fast estimation of Gaussian mixture models for image segmentation. Mach. Vis. Appl..

[28] Nainan S., Kulkarni V. (2021). Enhancement in speaker recognition for optimized speech features using GMM, SVM and 1-D CNN. Int. J. Speech Technol..

[29] Agarwal M., Rao K.K., Vaidya K., Bhattacharya S. (2021). ML-MOC: Machine Learning (kNN and GMM) based Membership determination for Open Clusters. Mon. Not. R. Astron. Soc..

[30] Rathnamala S., Jenicka S. (2021). Automated bleeding detection in wireless capsule endoscopy images based on color feature extraction from Gaussian mixture model superpixels. Med. Biol. Eng. Comput..

[31] Zhang R., Gong W., Grzeda V., Yaworski A., Greenspan M. (2013). An Adaptive Learning Rate Method for Improving Adaptability of Background Models. IEEE Signal Proc. Let..

[32] Rong D., Wang H., Xie L., Ying Y., Zhang Y. (2020). Impurity detection of juglans using deep learning and machine vision. Comput. Electron. Agric..

[33] Tanjung J.P., Muhathir M. (2020). Classification of facial expressions using SVM and HOG. J. Inform. Telecommun. Eng..

[34] Deore S.P., Pravin A. (2019). Histogram of Oriented Gradients Based Off-Line Handwritten Devanagari Characters Recognition Using SVM, K-NN and NN Classifiers. Rev. Intell. Artif..

[35] Setiawan A., Diyasa I.G.S.M., Hatta M., Puspaningrum E.Y. (2020). Mixture gaussian V2 based microscopic movement detection of human spermatozoa. Int. J. Adv. Intell. Inform..

[36] Kavitha K., Tejaswini A. (2012). VIBE: Background detection and subtraction for image sequences in video. Int. J. Comput. Sci. Inf. Technol..

[37] Deng J., Sun W., Guan L., Zhao N., Abbasi Q.H. (2019). Noninvasive Suspicious Liquid Detection Using Wireless Signals. Sensors.

[38] Ji L., Lin M., Jiang W., Cao G., Xu Z., Hao F. (2022). An improved rock typing method for tight sandstone based on new rock typing indexes and the weighted fuzzy kNN algorithm. J. Petrol. Sci. Eng..

[39] Abu Alfeilat H.A., Hassanat A.B., Lasassmeh O., Tarawneh A.S., Alhasanat M.B., Eyal Salman H.S., Prasath V.S. (2019). Effects of distance measure choice on k-nearest neighbor classifier performance: A review. Big Data.

[40] Wu D., Wu Q., Yin X., Jiang B., Wang H., He D., Song H. (2020). Lameness detection of dairy cows based on the YOLOv3 deep learning algorithm and a relative step size characteristic vector. Biosyst. Eng..

[41] Lawal M.O. (2021). Tomato detection based on modified YOLOv3 framework. Sci. Rep..

[42] Knapp J.Z., Abramson L.R. (1990). Automated particulate inspection systems: Strategies and implications. PDA J. Pharm. Sci. Technol..

[43] Wang Y., Zhou B., Zhang H., Ge J. (2012). A vision-based intelligent inspector for wine production. Int. J. Mach. Learn. Cybern..

[44] Wang Y., Ge J., Zhang H., Zhou B. (2011). Intelligent injection liquid particle inspection machine based on two-dimensional Tsallis Entropy with modified pulse-coupled neural networks. Eng. Appl. Artif. Intel..

[45] Mahfouf Z., Merouani H.F., Bouchrika I., Harrati N. (2018). Investigating the use of motion-based features from optical flow for gait recognition. Neurocomputing.

[46] Zhou B., Wang Y., Ge J., Zhang H. A machine vision intelligent inspector for injection. Proceedings of the IEEE Pacific-Asia Workshop on Computational Intelligence and Industrial Application.

[47] Barnich O., Van Droogenbroeck M. (2011). ViBe: A Universal Background Subtraction Algorithm for Video Sequences. IEEE Trans. Image Process.

[48] Stauffer C., Grimson W.E.L. Adaptive background mixture models for real-time tracking. Proceedings of the IEEE Computer Society Conference on Computer Vision and Pattern Recognition (Cat. No PR00149).

[49] Huang B., Ma S., Lv Y., Liu C., Zhang H. (2014). The study of detecting method for impurity in transparent liquid. Optik.

[50] Ge J., Wang Y., Zhou B., Zhang H. (2009). Intelligent Foreign Particle Inspection Machine for Injection Liquid Examination Based on Modified Pulse-Coupled Neural Networks. Sensors.

